# Supplementary figures and images for: Exploring the ovine sperm transcriptome by RNAseq techniques. I Effect of seasonal conditions on transcripts abundance
Source: PLoS One. 2022 Mar 14;17(3):e0264978. doi: 10.1371/journal.pone.0264978 (PMC8920283; doi:10.1371/journal.pone.0264978)

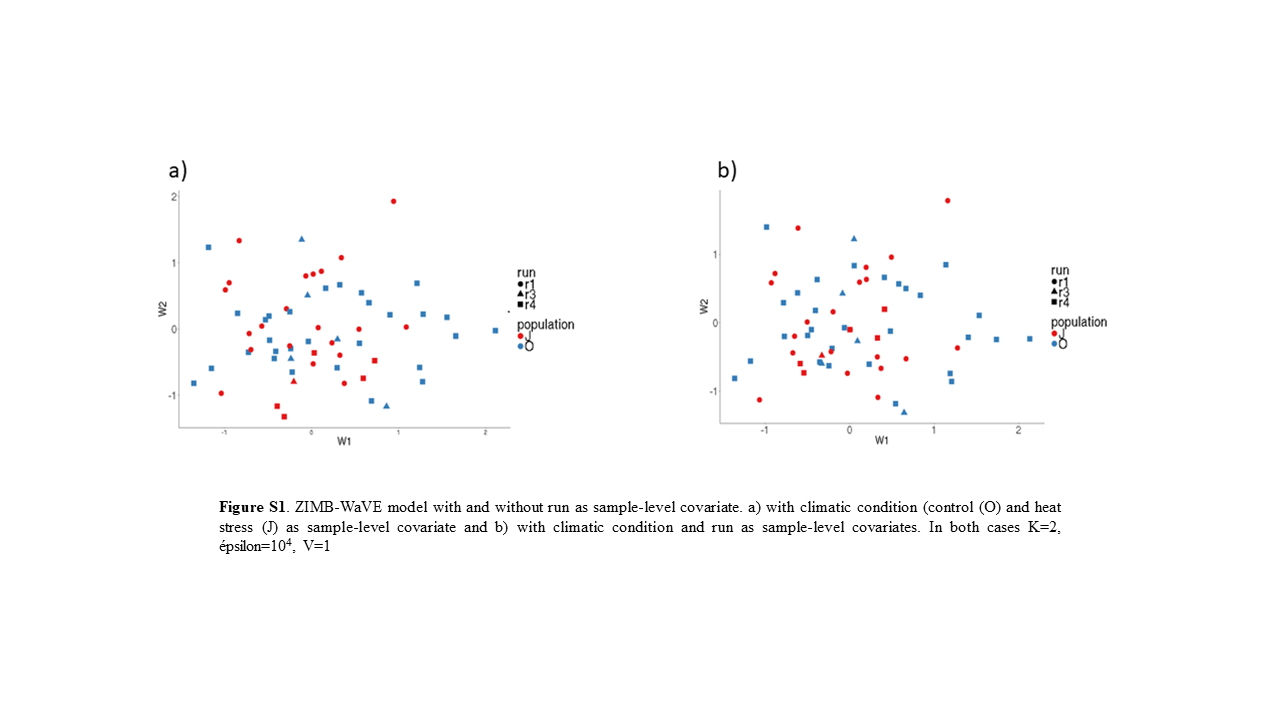

Supplement: S1 Fig — a) with climatic condition (control (O) and heat stress (J) as sample-level covariate and b) with climatic condition and run as sample-level covariates. In both cases K = 2, épsilon = 104, V = 1. (TIF) [file pone.0264978.s001.tif]
