# Supplementary material for: Exploring the ovine sperm transcriptome by RNAseq techniques. I Effect of seasonal conditions on transcripts abundance
Source: PLoS One. 2022 Mar 14;17(3):e0264978. doi: 10.1371/journal.pone.0264978 (PMC8920283; doi:10.1371/journal.pone.0264978)
Supplement: Attachment — Submitted filename: Response to reviewers_13-01-2022.docx [file pone.0264978.s009.docx]

**Response to Reviewers**

**Journal Requirements:**

**1. Please ensure that your manuscript meets PLOS ONE's style requirements, including those for file naming. The PLOS ONE style templates can be found at https://journals.plos.org/plosone/s/file?id=wjVg/PLOSOne_formatting_sample_main_body.pdf and https://journals.plos.org/plosone/s/file?id=ba62/PLOSOne_formatting_sample_title_authors_affiliations.pdf**

Response: We have upgraded the manuscript formatting based on the requirements (e.g. Figure has been replaced by Fig; Supplementary Legends tittles are now in bold).

**2. Thank you for stating the following in the Acknowledgments Section of your manuscript:**

**"RTA2013-00041 INIA project has provided the funding to do this work."**

Response: Ok. Sorry for the mistake. Funding-related text has been removed from the manuscript. For this work we declare that "The authors received no specific funding for this work", as figure in the online submission. In the ACNOWLEDGEMENTS section we have add the following sentence: “This work has been supported by the RTA2013-00041 INIA project”.

**Reviewer #1: In this study, Urena et al., reported RNA-seq results of ovine sperm transcriptomes from 64 ejaculates. Among them 28 were collected in summer and 36 were collected in autumn, allowing them to evaluate the seasonal effects on the sperm transcriptome. The authors performed functional enrichment analyses on total sperm transcripts, as well as on differentially abundant (DA) transcripts, and identified several enriched functional categories. Once published, these sequencing data will provide the community with very useful resources of ovine sperm RNAs. However, before that, I have some questions and comments listed below.**

**1. I didn’t find ‘figure legends’ in the manuscript, which made it hard for me to understand each figure.**

We apologize, there must have been some error during the uploading of the manuscript. Figure legends have been added to the main manuscript after the paragraph in which they are first cited.

**2. Figure 1 looks confusing. The numbers from the pie chart didn’t add up to the number(s) of protein coding genes / transcripts shown at the bottom-right corner. Did the authors happen to exclude the largest category, the protein-coding mRNAs?**

Thanks for pointing out this mistake. Percentages for the different kind of genes/transcripts in Figure 1 were indeed wrong. They were calculated without taking into account the total genes detected. An updated Figure 1 has been created. Protein coding genes account for the 91% of the total genes/transcripts detected, and the remaining (approx. 9%), corresponded to miRNA, miscRNA, Mt rRNA, snoRNA, etc. We have included the acronyms meaning in the figure legend.

**3. For Figures 2 and 5, if GSEA results are shown here, it would be better to show NES (Enrichment score) and p value, rather than the bar plots of percentages. Also, without figure legend, I don’t understand what the percentages mean.**

We have updated Figures 2 and 5 as the reviewer suggested. In the new figures, additional tables with GO terms IDs and names, number of genes in each GO term, NES values, nominal p_values and FDR values have been included.

**4. The raw sequencing data need to be made publicly available upon publication of the study. The authors can upload the data to databases such as ‘Gene Expression Omnibus (GEO)’, and add a paragraph of ‘Data Availability Statement’ to the manuscript.**

Raw data of this work has been submitted to the NCBI repository with the Bio Project accession number PRJNA733107. We have included a “Data availability” statement after the supplementary material legends:

L1099-1100: “Data availability: The datasets generated and analysed in the current study are available at NCBI’s BioProject PRJNA733107.

**5. The authors only identified 98 differentially abundant genes, by using the criteria of Padj < 0.05 and Log2FC >= 1.5. (I am not sure if I understand correctly, as in L263 the authors wrote >= 1.5, but in L268 they wrote >= 2.0. Please double check).**

**Here, the cutoff for fold changes is pretty harsh (2^1.5 = 2.83 fold). I would suggest use a milder cutoff, for example Log2FC >=1 so that’s 2 fold. In this case the authors may get more DA genes and make more sense of the functional differences. But this is optional.**

Thank you for your comments. In the case of sperm transcriptome and since the difficulties to detect differences in gene expression we have decided to choose a high logFC threshold to avoid having false positive DA genes. We preferred to have less DA genes, but real, than higher DA genes that could include several false positives that could hamper the real biological effects.

Sorry for the mistake. Effectively, we have considered the threshold log2FC > 1.5 to declare a gene as DA. However, in Figure 4 we only showed those DA genes with a log2FC > 2, but only for a practical question, and that is that they could be represented graphically, otherwise the Figure was too big. We are making this clear in the Figure 4 legend:

*Fig 4. Upregulated (dark grey) and down-regulated (light grey) protein coding genes under heat stress conditions. The DA genes passed the filters of padj < 0.05 and absolute log2FoldChange ≥ 1.5. In this figure, we only show genes that presented padj < 0.05 and absolute log2FoldChange ≥ 2.”*

**Reviewer #2: In this submission, a study of the sperm ovine transcriptome has been developed in the view to get knowledge of the different sperm RNAs abundances and their potential modifications during heat variation between summer and autumn.**

**An important sequencing analysis of ram sperm RNAs of 40 animals is presented with differences according to the temperature of the environment surrounding of the animals. More than 10 000 transcripts have been obtained and 236 genes were differently expressed between summer and autumn**

**General comments: Important results about the sperm RNAs are presented in this submission. The analysis of the results has been done with probably the best methods but a lot of information is need.**

**First of them is relative to the characteristic of the sperm collected for this analysis before purification. Nowhere information is given such as concentration, morphology, mobility, presence or not of the cytoplasmic droplets etc. for the samples obtained in July and October.**

Sperm characteristics of the ejaculates collected in these rams for this work, are those routinely measured in the artificial insemination centre. These sperm quality traits were ejaculate volume, the spermatozoa concentration and mass motility (evaluated on a 0 to 5 scale). No data about morphology and cytoplasmic droplets have been collected. All ejaculates used in this study met the quality requirements set at the centre for use in AI.

**Furthermore, as these samples came from an artificial insemination centres, fertility index of the animals during summer and autumn should be probably presented between these two seasons.**

Effectively data of the rams came from an artificial insemination centre. The rams used in this work are those used for inseminate ewes in all the herds participating in the genetic breeding program of this breed to improve milk traits. However, since to perform this work we used the complete ejaculates of this rams in each season, no data about the fertility index of these ejaculates are available, since no inseminations were performed with them. However, if the reviewer feels it is important to indicate the raw fertility values on each season, we could request such information from the breeders association and the AI centre.

**The choice of the two seasons is based on the temperature but what about the variation of the endocrinology of the animals induced with the photo period?**

Regarding the endocrinology changes induced as effect of the photoperiod, no measures have been made in this work. It is important to remark that the biological material used in this work came from commercial rams which belonged to the artificial insemination centre and only one ejaculate was provided. Therefore, it is not possible to take and analyse different measures from those routinely collected in the centre.

**The sequencing methodology is unclear. The RNAs from 24 animals have been sequenced but how the analysis has been done? Each couple of sperm samples has been analysed by animal or a mix has been done for each season? What is the variation of the results between animals? How much RNAs are common between animals?**

The analysis to test differential RNA abundance in July and October samples consisted of a comparison between individual samples taken in each season but including the animal effect to take into account that most samples were taken in the same experimental unit (the animal) and therefore a common environment effect must be considered. . Including this information in the model design will account for differences between the samples while estimating the effect due to the heat conditions. To make it more clear, we now include in the material and methods of this analysis:

Page 6, lines 207-209: *“The effect of the animal was also included in the DESEq2 model using a multi-factor design which includes sample information.”*

**Information of the length of the sequence should be given somewhere.**

Characteristics relative to RNA sequencing are fully detailed in S1 Table. HighSeq2000 Illumina platform yields 75bp long paired-end reads. In S1 Table and for each RNA sequence run (OVISPERM_01, 03 and 04) the column "Avg alignment insert size" shows the average sequence length for each sample.

**In the discussion, the variations of the RNAs should be discussed with the parameters of the sperm characteristics.**

We understand the interest of the reviewer in the analysis of the association between transcripts abundance and sperm characteristics. But, as the title of the work reflects – “Exploring the ovine sperm transcriptome by RNAseq techniques. I Effect of climatic conditions on transcripts abundance”- this is the first manuscript in a series of three regarding a central topic that is the study of the changes in the ram’s sperm transcriptome and metagenome due to climatic effects and their impact in sperm characteristics linked to their reproductive efficiency.

In this large experiment we have collected not only sperm samples from rams in two different seasons but a few sperm quality traits, volume and concentration, and assessed sperm DNA fragmentation assessed by flow cytometry for all the samples collected. The aim of the second part of this work is to analyse the relationship between transcripts abundance and sperm characteristics and also, a variant calling study with the aim to detect variants affecting transcripts abundance in both seasonal conditions. As the part I of this work is already very long, we considered necessary to split the work into three distinct parts. The first that currently concerns us, a deep and discussed analysis of the ram’s transcriptome and the effect of climatic conditions on transcripts differential abundance. For example, this type of work alone has already been published in other species, as in boars, “A RNASeq Analysis to Describe the Boar Sperm Transcriptome and Its Seasonal Changes” Gòdia et al., 2018, and it has helped on later studies related to sperm quality parameters, to discern between real differently abundant genes than from those false positives, identified by differences of heat stress. In the second study, we aim to perform an association analysis between transcripts abundance and sperm traits, and perform a variant calling analysis. And the third, we aim to study of the microbiome transcripts existing in sperm samples and its changes due to seasonal changes.

This is the reason for not including this data, because we want to prepare a second manuscript with the association of transcripts abundance and sperm characteristics as the reviewer very well suggests it. But we prefer to split it, to ensure that the message of the role of heat stress in the ovine transcriptome is clear. We have identified several transcripts altered because of heat stress, and we feel is important for the scientific community to share this results for potential future work on this field.

**Ref 26 is not associated with translational activity IN the sperm cells.**

Sorry for the mistake. This reference is wrong. We have included the correct reference in the References section.

*26	Gur Y, Breitbart H. Mammalian sperm translate nuclear-encoded proteins by mitochondrial-type ribosomes. Genes Dev. 2006; 20(4):411-416. doi:10.1101/gad.367606*
