# Supplementary material for: Exploring the ovine sperm transcriptome by RNAseq techniques. I Effect of seasonal conditions on transcripts abundance
Source: PLoS One. 2022 Mar 14;17(3):e0264978. doi: 10.1371/journal.pone.0264978 (PMC8920283; doi:10.1371/journal.pone.0264978)
Supplement: Attachment — Submitted filename: Response to reviewers_07-02-2022.docx [file pone.0264978.s010.docx]

**Response to Reviewers**

**Additional Editor Comments:**

**One reviewer is only partially satisfied with the proposed revised version. The reviewer raises a major concern that should be appropriately addressed. Currently, this is not the case. Please take this into account and modify your report accordingly. This will be the last time you have the opportunity to respond to the reviewer's comment.**

We would like to thank you for the time and effort in reviewing this work, and for the suggestions made by the reviewers. We also thank the editor for the opportunity to clarify the doubts raised by reviewer 2. We hope that both, the reviewer and the editor, agree with our responses, and if there are no other major concerns, the article will be accepted for publication.

**Reviewer #2**

**According to my comments about the lack of information on the spermatozoa parameters of the samples used for this transcriptomic study, the authors written that these parameters are those of artificial centre. However, according to the authors, these parameters should be given in second submission. How could a reviewer and a reader interpret such episode publications? It should be better that these publications should be submitted in same time.**

We agree with the reviewer that in addition to the characterization of the sheep sperm transcriptome and its seasonal variation, the link of these RNA changes with sperm quality traits is of high interest due to their relevance for the sheep production sector. To help understanding the relevance of our results, we have modified the article accordingly and ***have now added a table (Table 2) showing the average and standard deviation values for the different semen parameters in each group and also the results of a T-test comparing these values in both groups***. The results show that the ejaculate concentration was significantly different in heat vs comfort samples, but not the rest of the traits. This may mean that transcriptional changes between seasons are efficient in protecting sperm cells from possible damage caused by environmental changes, so that sperm characteristics are little affected by them Therefore, the identification of the genes (and the metabolic routes in which they are involved) that show significant changes in their RNA abundance in sperm to cope with seasonal changes is an important step to disentangle its potential effect on sperm characteristics, and by extension, on the reproductive efficiency of the males.

We believe that evaluating the seasonal differences of the ovine sperm transcriptome is of relevance, because it can highlight the crucial genes that cope during changes in environmental conditions during spermatogenesis to grant the efficient production of viable and functional spermatozoa. We are tackling this question from two different angles. One angle, addressed in this article, is to understand the molecular changes that may help coping with seasonal environmental variation. The other angle is to directly relate the abundance of each gene in sperm, with the semen quality in relation to these seasonal changes. We are currently drafting a second manuscript tackling this other angle.

**Furthermore, all the discussion of the present submission is based on a heat effect but nowhere the condition of the ram breeding was given. Are the animals in the barn or in open air? Is the temperature given in the text the same in the barn etc.?**

The rams evaluated in this study were kept in standardized conditions in the artificial insemination centres. All the animals were in conditioned boxes within the barn, with natural ventilation and with similar feeding and handling. The animals are allowed to graze outdoors daily, but avoiding the hottest hours during the summer months. The station itself does not have a weather station. Thus, the meteorological conditions that we reported were provided by the closest official meteorological station which shares very similar weather conditions for the short distance and similar altitude. Importantly, this data indicates clear differences in temperature and relative humidity between the hot and the comfort seasons. A paragraph providing this information has been added to the manuscript.

**Page 2, lines 95-98: *“). Rams were handled in conditioned boxes inside the barn, with natural ventilation and similar feeding and management. The animals are allowed to go outside the stables daily, but avoiding the hottest hours of the summer months. The temperature recorded (Table 1) reflects the outdoor conditions, in order to display the differences between the hot and comfort seasons”.***

**Numerous publications shown that the temperature regulation of the testis of the ram could be very efficient even in hot environment. A temperature effect is probable but could not presented in this submission has the only effect on the transcriptome result. The sexual activity of the ram is completely different between the summer and autumn (androgen, sperm production and quality etc.).**

We fully agree with this statement, and even more so for this breed of sheep which is very well adapted to the climatic conditions of this particular geographical region. This efficient regulation at the physiological and anatomical levels, arises from the fact that in most mammals the testicle, which is contained in the scrotum, is exposed to a temperature that is approximately 3°C lower than the inside body temperature (***Hansen PJ. Philos Trans R Soc Lond B Biol Sci. 2009; 364(1534): 3341-50***) to protect the sperm cells from heat injuries. However, despite the existence of this efficient temperature regulation in the mammalian testicle, the high sensitivity of sperm cells to environmental heat has been extensively accepted and studied (***Perez-Crespo M et al. Mol Reprod Dev. 2008; 75(1):40-7; Grazer VM, Martin OY. Biology (Basel). 2012; 1(2):411-38; Takahashi M., Reprod Med Biol (2012) 11:37–47; Durairajanayagam D. Reproductive BioMedicine Online Vol. 2014, Vol 30(1) p14-27; Shahat A.M. et al. Theriogenology 158 (2020), 84-96***). Heat stress arises when the effective temperature of the environment exceeds the animals’ upper critical temperature, which in sheep ranges between 25 and 31 °C, depending on breed, age and physiological state (***Hopkins PS et al. Aust J Agric Res. 1978; 29:161–71***). The physiological stress caused by hot weather can alter the sperm’s transcriptome (***Jannatifar R. et al. Association of heat shock protein A2 expression and sperm quality after N-acetyl-cysteine supplementation in astheno-terato-zoospermic infertile men. Andrologia. 2021 Jun; 53(5):e14024;*** ***Lymbery RA. Et al. Post-ejaculation thermal stress causes changes to the RNA profile of sperm in an external fertilizer. Proc Biol Sci. 2020 Nov 11; 287(1938):20202147; Rizzoto G. et al. Acute mild heat stress alters gene expression in testes and reduces sperm quality in mice. Theriogenology. 2020 Dec; 158:375-381***) and even the semen quality and fertility of the animals (***Jeremy M. et al. Co-treatment of testosterone and estrogen mitigates heat-induced testicular dysfunctions in a rat model J Steroid Biochem Mol Biol. 2021 Nov; 214: 106011; Flowers WL. Factors affecting the production of quality ejaculates from boars. Anim Reprod Sci. 2021 Sep 6:106840; Pirani M. et al. Protective Effects of Fisetin in the Mice Induced by Long-Term Scrotal Hyperthermia. Reprod Sci. 2021 Nov; 28(11):3123-3136; Llamas-Luceño N. et al. High temperature-humidity index compromises sperm quality and fertility of Holstein bulls in temperate climates. J Dairy Sci. 2020 Oct; 103(10):9502-951***). The scientific literature suggests that to cope with environmental stressors there is a regulation of the expression of certain genes involved in the heat-stress response (***Godìa M, et al. Front Genet. 2019; 10:299; Ramon M, et al. PLoS One. 2014;9(1):e86107. Salces-Ortiz J et al. PLoS One. 2015; 10(2):e0116360. Hansen PJ. Philos Trans R Soc Lond B Biol Sci. 2009; 364(1534):3341-50***).

It is true, as reviewer 2 pointed out, that there are other factors related with the sexual activity of rams along seasons. For example, the photoperiod has an important impact in this sexual activity as it is related to the seasonal anoestrus. However, in medium-latitude regions, commonly found in regions with mild winters, as is the case of the region in the Iberian Peninsula described in our study, the fertility of rams shows less seasonal breeding behaviour affected by the photoperiod; thus, testicular size, sperm production, and mating capacity are mildly reduced during the increased photoperiods (***Avdi et al. 2004, Theriogenology, 62, 275–282***). Our data regarding sperm quality traits (Table 2) shows that that there were no differences between them due to seasonal changes, except for sperm concentration. Therefore, the potential effect of the photoperiod on the reproductive efficiency of the Manchega rams in our geographical location, and reproductive systems with artificial insemination, is likely to be small. Likewise, the effect of the photoperiod in the transcriptome changes that we observed in our study should be low.

Regarding this topic we have added in the conclusions of the manuscript the following sentence:

**Pages 18-19, lines 724-732. *“In this work, 80 ejaculates from 40 rams of the Spanish Manchega breed, collected in two seasons with differential climatic conditions (heat and comfort) were used to assess the core sperm transcriptome and its changes due to seasonal factors. In medium-latitude regions, as is the case of the region in the Iberian Peninsula described in our study, the fertility of rams shows less seasonal breeding behavior affected by the photoperiod [122]. Therefore, climatic factors must be the most important determinants of the changes observed in the sperm transcriptome. In this work we have found that despite the transcriptional inactivity of sperm cells, rams spermatozoa carry thousands of RNAs which are not merely residual products from the spermatogenesis process, but have critical functions for the spermatozoa functionality and surviving and for egg fertilization and embryo development”*.**

**I suggest also that the title should be:” Exploring the ovine sperm transcriptome by RNAseq techniques. I Effect of seasonal conditions on transcripts abundance”.**

We fully agree with the opinion of reviewer 2 regarding the title of the manuscript. We have changed the title of the manuscript to the one rightly suggested by reviewer 2.

***New title of the manuscript:” Exploring the ovine sperm transcriptome by RNAseq techniques. I Effect of seasonal conditions on transcripts abundance”.***
